# Exploring Proteins in *Anopheles gambiae* Male and Female Antennae through MALDI Mass Spectrometry Profiling

**DOI:** 10.1371/journal.pone.0002822

**Published:** 2008-07-30

**Authors:** Francesca R. Dani, Simona Francese, Guido Mastrobuoni, Antonio Felicioli, Beniamino Caputo, Frederic Simard, Giuseppe Pieraccini, Gloriano Moneti, Mario Coluzzi, Alessandra della Torre, Stefano Turillazzi

**Affiliations:** 1 Centro Interdipartimentale di Spettrometria di Massa, Università degli Studi di Firenze, Firenze, Italy; 2 Dipartimento di Anatomia, Biochimica e Fisiologia Veterinaria, Università di Pisa, Pisa, Italy; 3 Sezione di Parassitologia, Dipartimento di Scienze di Sanità Pubblica, Università ‘La Sapienza’, Roma, Italy; 4 Institut de Recherche pour le Développement, Montpellier, France; 5 Organisation de Coordination pour la Lutte contre les Endémies en Afrique Centrale, Yaoundé, Cameroun; University of Helsinki, Finland

## Abstract

MALDI profiling and imaging mass spectrometry (IMS) are novel techniques for direct analysis of peptides and small proteins in biological tissues. In this work we applied them to the study of *Anopheles gambiae* antennae, with the aim of analysing expression of soluble proteins involved in olfaction perireceptor events. MALDI spectra obtained by direct profiling on single antennae and by the analysis of extracts, showed similar profiles, although spectra obtained through profiling had a richer ion population and higher signal to noise ratio. Male and female antennae showed distinct protein profiles. MALDI imaging experiments were also performed and differences were observed in the localization of some proteins. Two proteins were identified through high resolution measurement and top-down MS/MS experiments. A 8 kDa protein only present in the male antennae matched with an unannotated sequence of the *An. gambiae* genome, while the presence of odorant binding protein 9 (OBP-9) was confirmed through experiments of 2-DE, followed by MS and MS/MS analysis of digested spots. This work shows that MALDI MS profiling is a technique suitable for the analysis of proteins of small and medium MW in insect appendices, and allows obtaining data for several specimens which can be investigated for differences between groups. Proteins of interest can be identified through other complementary MS approaches.

## Introduction

For their survival and reproductive success, mosquitoes depend on a series of behaviours such as mating, foraging and oviposition, which are modulated by internal and external cues. Olfactory cues are the most important group of external stimuli affecting mosquito behaviour, such as mating and partner recognition [1]. Moreover, males also respond to odours from plants that represent their only feeding sources, while several female behaviours, ranging from host-seeking to oviposition, are mediated by odour cues originating from their hosts. Perception of volatile semiochemicals in mosquitoes is mediated, as for other insects, by chemosensory neurons segregated within specific olfactory sensilla located mainly on the antennae and maxillary palps. Analogously to the vertebrate olfactory system, the detection of odour molecules involves Odorant Binding Proteins (OBPs), soluble proteins very abundant in the antennal chemosensilla [2]. These proteins are believed to carry the odour molecules from the porous cuticular surface of the antennal sensilla through the sensillar lymph to the G-protein-coupled odorant receptors residing on the olfactory sensory neurons [2].

Several studies have recently been focused on the mechanisms of semiochemical perception in Anopheline mosquitoes and on the characterization of molecules implicated in the olfactory signalling pathways see [3], [4]. In fact, the high anthropophily of major malaria vectors, such as some of the afro-tropical species of the *Anopheles gambiae* complex, is recognised to represent one of the behavioural traits mostly responsible for their high vectorial capacity. It has, thus, been proposed that a better understand of the molecular mechanisms of the host-seeking behaviour would possibly allow the development of novel tools for malaria control, through the reduction of man-vector contact and/or a shift in its host-preferences [4]. Based on the published genome of *An. gambiae*, 57 genes encoding for putative OBPs have been identified in this species [5], and classified into three different groups, the “classical OBPs”, the “atypical OBPs” and the “Plus C-OBPs”. The differential expression of these genes has been analysed in the two sexes through RT-PCR and microarrays [4], [6], and differential expression has been found for some classical OBPs and Plus-C OBPs.

Matrix assisted laser desorption ionization (MALDI) is a mass spectrometry (MS) technique widely used in proteomic studies, which has been successfully applied also to the direct analysis of peptides and low and medium molecular weight proteins in biological tissues, single cells and down to single organelles [7]–[10].

Although MALDI imaging and profiling MS have been applied mostly to the analysis of proteins in mammalian tissues [10], profiling has also been successfully used to study the spatial distribution of neuropeptides in sea hares (*Aplysia*), in crabs, as well as in insects reviewed in [11]. Through this approach, Rubakin and co-workers have analysed the content of neuronal vesicles of *Aplysia californica* [8] and localised the distribution of peptidic messengers in different regions within a single neuron [9]. A similar approach has been used by Yew and corworkers [12] to study neuropeptides in the nematode *Ascaris suum*.

In insects, MALDI profiling MS has been used by Clynen and coworkers [13] to analyse peptides in the complex formed by the pars intercerebralis and corpus cardiacum in *Locusta migratoria*. Larval peptidic hormones of *Drosophila melanogaster* were studied by Wegener and co-workers through MS profiling and MS/MS experiments directly on the neurohaemal organs [14]. More recently both MALDI imaging MS and MS/MS experiments were also successfully performed on neuropeptides of the house cricket *Acheta domesticus* [15]. The same paper also reports MS/MS experiments aimed at analyzing the spatial distribution of phospholipids in the *corpora cardiaca* and *corpora allata* of the same species.

The possibility to statistically compare MS spectra obtained from different anatomical areas or from diseased and healthy tissues through profiling or imaging experiments has recently been used in biomedical research, mainly with the aim of identifying biomarkers [16], [17]. A similar approach has never been reported in studies on insect tissues; however it could be used for studying protein presence in different tissues or organs of the same individual, in the same organs of individuals of different sexes or morphs, and in the same organs of individuals belonging to closely related species. Comparisons between closely related insect species have used MALDI MS spectra on non-tissue biological samples, but generally lacked statistical analysis [18]–[19]. To our knowledge, only the study by Turillazzi et al. [20] on the protein fraction of the venom of different wasps included a statistical analysis and demonstrated consistent differences between closely related species.

In the present work we used MALDI profiling coupled with a statistical approach, to detect small/medium proteins in the antennae of the major malaria vector species, *Anopheles gambiae* sensu stricto (s.s.), and we compared the mass spectra profiles obtained for the two sexes. With a view of exploring whether MALDI imaging MS could be applied to the analysis of protein distribution in insect appendices, imaging experiments were also attempted on the antennae of both female and male specimens. As a proof of principle, the presence of one OBP identified through the MALDI MS approach was also confirmed through the classic MS and MS/MS analysis of tryptic digest from 2-dimensional electrophoresis spots.

## Materials and Methods

### Chemicals

Acetonitrile, methanol, ethanol and deionized water were of LC-MS grade and purchased from Baker (Milan, Italy). Formic and trifluoroacetic acid (TFA) were respectively purchased from Baker and Sigma (Milan, Italy). Sinapinic acid was high purity grade and purchased from Sigma.

2DE Chemicals. IPG strips, IPG buffer, acrylamide and molecular weight marker proteins were from GE Healthcare bio.sciences (Uppsala, Sweden). All other chemicals were from Sigma (St. Louis, USA).

## Methods

### Insect specimens

All the specimens analysed in the present work belonged to *An. gambiae* s.s. M molecular form [21]. Individuals of sample A, originated from a colony named GAMCAM originated from the progeny of females collected in Cameroon. All the specimens were 2 days old and both sexes were fed with sugar only. Specimens were killed by freezing at −20°C and kept at this temperature until analysis.

Specimens of sample B were collected at the larval stage in Leboudi, a district of Yaoundé (3°52′N; 11°31′E), the capital city of Cameroon, in April-May 2006. Larvae were transported to the laboratory, reared until emergence and killed by freezing at −20°C when they reached the second day of adult life. Frozen specimens were then sent to the Department of Public Health at the University of Rome “La Sapienza”, where they were identified at the molecular form level by RFLP-PCR directly performed on a single wing [22].

### Protein extraction from antennae

Antennae were dissected from the frozen specimens under a binocular microscope with the aid of forceps and entomological pins. For sample A, protein extracts were prepared from a pool of 40 male antennae (20 specimens) and from a pool of 80 female antennae (40 specimens), being female antennae smaller and less plumose than males. Antennae were immersed in a 1∶1 methanol/0.1% TFA solution, sonicated in an ultrasonic bath for 30 min and then centrifuged for 5 min at 16,100 g. The supernatant was recovered, filtered through 0.2 µm pore size membrane (Minisart RC 15 Sartorius), dried in a speed vac centrifuge (RC 1010, ThermoElectron, Brema) and subsequently dissolved either in a 0.1% TFA (for MALDI-TOF MS analysis), or in a 0.1% FA (for LC/ESI-MS analysis) solution. In the case of the female extract, the final concentration was of two antennae/µL equivalent, while it was one antenna/µL for males.

### Imaging and profiling experiments by MALDI –TOF

Single antennae of *An. gambiae* males and females from sample A were attached to a conventional MALDI target using a double sided conductive tape (3 M Co). In the same way we dissected out and attached to the target, the mouth apparatus (proboscis and palps) and one single wing from some males and females. Matrix solution containing ethanol have been used with the aim of fixing tissues in MALDI MS experiments [10], although washing with fixing solvents before matrix application is preferred [23]. Since this latter method could not be applied to our samples due to their tiny dimensions, matrix solutions containing ethanol were tested. Eventually we used a solution of 20 mg/mL of sinapinic acid in acetonitrile, ethanol and 0.1% TFA (8∶46∶46). In comparison with conventional solution, containing only acetonitrile and TFA 0.1% as solvent, this mixture provided more intense spectra, possibly because of the partial removal of the epicuticular lipids by ethanol, which may favour the contact between the matrix and the sample proteins. When performing profiling experiments, the matrix was applied as discrete droplets by using a 2 ìl pipettor: 0.2 µl were deposited on a single antenna, dried and re-applied a second time. In the imaging experiments, the sample was spray coated by using a TLC glass reagent sprayer (Sigma). The MALDI target was held vertically about 20 cm from the sprayer nozzle and several applications were performed waiting ∼10 sec between two consecutive sprayings, until the antenna was homogenously covered with crystals, as observed by microscopic inspection.

In the profiling experiments on sample B, antennae were laid directly on the target and 1 µl of matrix solution was readily deposited on them. This allowed antennae to stick to the target.

Both profiling and imaging experiments were performed on a MALDI-TOF/TOF mass spectrometer Ultraflex I (Bruker Daltonics) by using Flex Control™ 2.4 and Flex Imaging 2.0 ™ as data acquisition software. Positive ion spectra were acquired in linear mode, setting the Ions Source 1 at 25 kV, the Ion Source 2 at 23.40 kV, the lens at 6.50 kV, and the delay time at 150 ns. The laser spot diameter was 100 µm.

Profiling experiments on sample B were performed by manually acquiring and accumulating mass spectra along the whole antenna. Fifty shots were collected for each spectrum and the final spectrum was generated by summing a total of 700 shots. Spectra acquired both in imaging and profiling experiments were later recalibrated according to the accurate molecular weight of three antennal proteins (8015, 11941 and 13936 Da), retrieved from analyses in liquid chromatography coupled to electrospray ionisation Fourier transform mass spectrometry (LC-ESI FTMS, see below).

In experiments on sample A, spectra were acquired over a 100×100 µm raster and for each position, either 60 or 100 shots were collected. When obtaining the final molecular images, normalization was applied using a Ymean/Ymax threshold of 0.02. The lower the value of this filter, the higher the number of noisy spectra were excluded from normalization. This value of the filter was chosen after a quick exploration of the mass spectra associated to the least intense ions on the image. Images were ultimately generated by setting for each considered m/z value the minimum intensity at 5% and the full intensity threshold at 100%.

### Comparison between sexes

In order to compare protein antennal profiles of males and females, the spectra obtained in profiling MS mode were imported into ClinProt Tools™ 2.0, Bruker Daltonics (CPT). The spectra were aligned setting the option maximum peak shift to 0.1% of the mass over charge (m/z) value. For each group, the program calculates an average mass spectrum as well as, for each ion signal, the probability (associated with the Student T and the Wilkoxon or Kruskall Wallis tests) that a determined peak has different intensity in different groups. Moreover, the program produces multivariate models highlighting the most discriminant signals for the separation between the groups. Only spectra acquired from sample B were submitted to statistical analysis, each spectrum being derived from one single specimen. In fact, in the case of sample A, more spectra were acquired from the same antenna; therefore within each sex, the spectra could not be considered as independent.

Similarly, we imported the spectra registered for the mouth apparatus, the wings, and the antennae originating from male specimens and compared the spectra registered for the three organs, without submitting the data to statistical analysis.

### Protein characterization by MALDI-TOF MS and LC-ESI FTMS

Proteins obtained from antenna extracts (see above) were analysed using the same MALDI-TOF mass spectrometer described above. In the case of male extracts, 1 µl of the sample was mixed to 1 µl of the matrix (sinapinic acid 10 mg/ml CH_3_CN∶0.1 TFA, 70∶40) on the target and allowed to dry. In the case of the female extract, 1.5 µl were mixed with 1.5 µl of matrix and applied to the target. Spectra were acquired in linear mode over the m/z range 5,000–20,000 for a total of 500 shots. The instrumental parameters were chosen by setting the ion source 1 at 25 kV, and the delay time at 80 ns. The instrument was externally calibrated prior to analysis using the Bruker protein calibrant kit (5000–17000 Da), and the sample spectra internally recalibrated according to the accurate molecular weight of three antennal proteins (8015, 11941 and 13936 Da) measured in the FTMS analyses. The male antenna extract was also submitted to HPLC-ESI FTMS analysis on a Ultimate 3000 (Dionex, San Donato Milanese, Italy) coupled to a LTQ Orbitrap mass spectrometer (Thermo, Bremen, Geemany) to determine the average and monoisotopic mass, and partial amino acid sequences through MS/MS experiments (top-down). A C_4_ capillary reverse phase column (15 cm×0.3 mm×5 µm, Vydac, Milan, Italy) was used. Proteins were eluted in a linear gradient ramping from 24% A to 55% B (A water, 0.1% formic acid; B 80% CH_3_CN; 20% water, 0.1% formic acid) in 30 min. Mass spectra were acquired in positive ion mode, setting the spray voltage at 2.80 kV, the capillary temperature at 280°C, the tube lens at 140 V and the FT nominal resolution (@ m/z 400) in MS scan at 60,000 and at 30,000 in MS/MS mode. The acquisition software (Xcalibur 2.0) was also set in data dependent scan, excluding mono- and doubly-charged ions from MS/MS experiments which were performed on the first and second most intense ions of the MS spectrum. Although the LTQ-Orbitrap mass spectrometer is designed for MS/MS experiments on peptides and small proteins, previous top-down experiments on isolated and abundant proteins belonging to the OBP family showed that spectra did allow obtaining short amino acid sequences.

Identification of proteins from 2-D gel spots. Antennae were dissected from 200 2-day-old males of the colony GA-CAM. Antennae were grounded in a mortar under liquid nitrogen. Proteins were extracted by using 250 µl of 50 mM Tris-HCl buffer pH 8 in presence of 40 µl of inhibitor Cocktail Solution (Sigma). After centrifugation at 19,000 g for 40 min, at 4°C, the supernatant was added with 105 mg of urea, 1.25 mg of CHAPS, 2 µl of Pharmalyte, 2 µl of IPG, 2 mg of DTT in order to obtain a rehydration buffer (8 M urea, 0.5% CHAPS, 1.6% IPG buffer, 1.6% Pharmalyte, 1% DTT), and a trace of bromophenol blue. 2-D gel separation was performed as described by Scarselli et al. [24]. Proteins in gel were detected by MALDI compatible silver staining and scanned with an Epson Expression 1680 Pro scanner.

Spots of interest were cut from the gel and destained. 40 µl of a 1 ng/µl of modified trypsin (Promega, Madison, WI) in 10 mM ammonium bicarbonate was added to each gel spot. After 40 min the solution was removed and substituted with a same volume of 10 mM ammonium bicarbonate only. Digestion was performed overnight at 37°C. Supernatants were recovered and digestion was blocked by adding 10% TFA. Peptides were separated by HPLC on the Ultimate 3000 and analysed on the LTQ-Orbitrap. Peptides were eluted on a PepSwift monolithic PS-DVB column (200 µm I.D.×5 cm, Dionex) in a linear gradient ramping from 100% of A (water 0.1% formic acid) to 60% B (80% acetonitrile; 20% water, 0.1% formic acid) in 12 min. Mass spectra were acquired in positive ion mode setting the spray voltage at 1.80 kV, the capillary temperature at 250°C, the tube lens at 140 V. The LTQ-Orbitrap was set to work in data dependent mode, by acquiring a full range spectrum at 15,000 resolution from 500 to 2000 Th and MS/MS spectra for the three top ions. Monocharged ions did not trigger MS/MS experiments. The acquired data were analysed using Sequest (Thermo Fisher Scientific Inc.) against a database created by merging *Anopheles gambiae* protein sequences downloaded via ftp at http://www.ensembl.org/info/data/download.html. (Anopheles_gambiae.AgamP3.48.pep.all.fa.gz, and Anopheles_gambiae.AgamP3.48.pep.abinitio.fa.gz) together with all entries containing “keratine” and “trypsin” within Swissprot. Searches were performed (i) allowing up to four missed cleavage sites (ii) carbamidomethylation of cysteins and oxidation of methionins as variable modifications and (iii) 10 PPM of tolerance for the precursor ions and 0.5 AMU for the fragment ions.

## Results

### Profiling and imaging MALDI experiments

**Figure 1:**
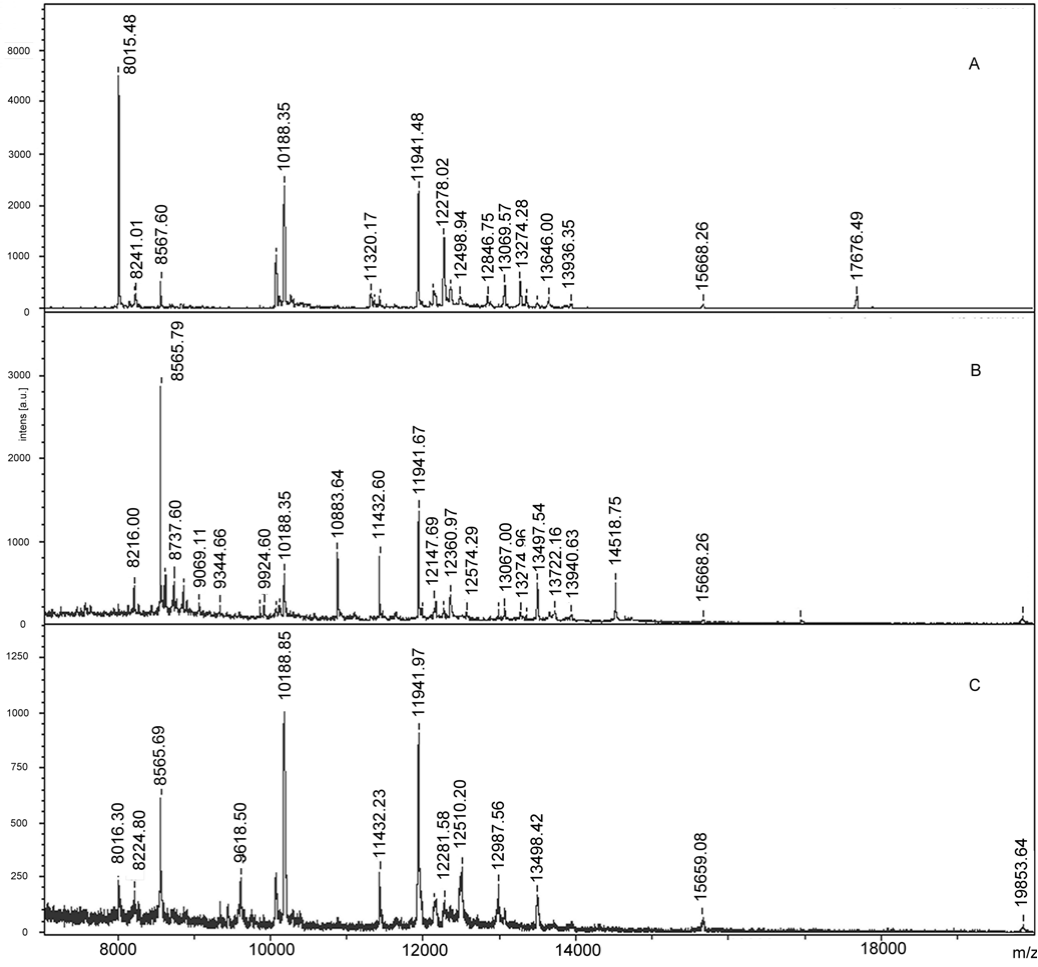
Direct MALDI profiling on *Anopheles gambiae* antenna. MALDI-TOF spectra obtained from direct profiling on a single antenna of male (A) and female (B) of *An. gambiae*. Panel (C) reports the spectrum obtained from the extract of female antennae; the extract volume analysed corresponded to the equivalent of three antennae.

Figure 1 (A and B) shows the mass spectra obtained from profiling experiments performed on antennae of both males and females mosquitoes (sample A). Mass spectra obtained from the MALDI MS analysis of antennal extracts showed the same pattern as those found in the profiling experiments, but the latter had a richer ion population and a higher signal to noise (S/N) ratio. The difference in the results obtained using the two approaches was particularly remarkable in the case of the females, where the direct profiling of one single antenna produced a satisfactory spectrum, whereas the spectrum obtained on the extract of 3 antennae equivalents exhibited signals with a lower S/N ratio (Fig. 1 C). No evident differences in spectra quality or intensity were observed between the experiments where antennae were attached using the conductive double sided tape or directly attached to the target with a matrix drop.

The spectra obtained in the imaging MS experiments were of variable intensity and quality, and in several cases we could observe a certain, even if small, shift between areas where signals were registered on the target and the location where the antennae were placed. This could be caused both by the matrix application (possibly generating delocalization) and by the thickness of the antenna. In the first case, although the matrix solution was carefully sprayed in order to avoid an excessive soaking of the sample, this precaution may have not been sufficient to hinder protein delocalization. With regard to the second aspect, both the presence of a greater thickness on the MALDI target due to the “rough” surface of the conductive tape and of the attached antenna, could affect laser focusing. Consequently the actual position where the laser beam hit the sample could have been slightly different from that dictated through the software, producing a “delocalization effect” on such tiny sample (about 1 mm in length). Nonetheless experiments produced spectra of good quality and although most proteins appeared to be similarly localised, some showed a different distribution across the antenna (fig. S1).

### Protein characterization by MALDI-TOF MS and LC-ESI FTMS

The identity of the proteins detected in first instance by direct profiling was further investigated by analysing the antennal protein extracts by MALDI-TOF MS. Having observed again the same species at the same MW, an LC-ESI FT MS analysis was performed and the accurate masses compared with the ones predicted for *An gambiae* OBPs reported in the literature. MS/MS top-down experiments were also run in an effort to identify the observed proteins.

The mass signal registered at 13,936 Th in the MALDI-TOF MS antennal profiling, and with an average mass of 13,935.74 Da in the protein extracts analysed through LC-ESI FTMS (fig.S2 A, B, C) was closer to the MW of AGAM OBP-9 (**AY146740.1**, mature protein with all six cysteine residues linked through disulphide bridges 13,935.70 Da) rather than to the MW of AGAM OBP-6 (**AF437889.1** ) if the signal peptide ranged from aa 1 to aa 33, as predicted by SignalP 3.0 server (http://www.cbs.dtu.dk/services/SignalP/; mature protein 13,935.09 Da) rather than from aa 1 to 34 as originally reported [6]. The identification of the protein as OBP-9 was also supported by the measure of the monoisotopic mass (13,926.71 Da fig. S2 D, the theoretical monoisotopic masses of OBP9 and OBP6 being respectively 13,926.77 and 13,925.80 Da respectively) and by the top-down experiments, which produced a spectrum compatible with the N terminal EFVV stretch of OBP9 (see supplemental material Text S1 and fig. S2 E). Moreover the OBP9 (AGAP000278-PA) was also identified in the 2D gel on the basis of the MS/MS spectrum arising from the double charged ion of the 1300.67 Da peptide matching the sequence DANEVREEIVK (with a probability of 1.3×10^−4^ that this match occurred by chance within the given database).

The mass signal registered at 8,015 Th in the MALDI-TOF profiling experiment was investigated as it discriminates between sexes (present in the males, absent in the females, see below). The monoisotopic mass of this was registered at 8,010.53 Da. The top-down experiment (fig. S3) performed on the parent ion at 1,604.12 Th (z = 5) produced a spectrum compatible with the stretch YSG(I/L)GYGYN. Blasting (http://130.14.29.110/BLAST/) these and their reverse sequences, using the option “Search for short, nearly exact matches” against the *Anopheles* genome, we found a hit (**gi|118781948**) for the YSGLGYGYN sequence (Y32-N40). Assuming a cleavage of the corresponding polypeptide between aa 29 and 30, the mature protein including aa 30–108 would have a theoretical monoisotopic mass of 8,010.57 Da in agreement with our experimental measure. Blasting this sequence against Swissprot database did not produce any result.

The ion signal registered as 12,846 Th in the MALDI-TOF MS spectra of male antennae was in agreement with the MW of OBP-10 (**AY146741**, mature protein with all cystein residues linked through disulphide bridges 12,847.94 Da). However the LC-ESI FTMS analysis measured this protein with an average and monoisotopic MW of 12,845.34 and 12,837.32 Da respectively (vs a theoretical monoisotopic value of 12,839.44), indicating that either this signal does not correspond to OBP-10 or amino acidic substitutions have occurred. Unfortunately top-down experiments on this protein did not produce reliable sequence information.

The signal observed at 14,518 Th in the MALDI-TOF profiling on female antennae could correspond to OBP-1 (**AY146721**, theoretical average mass 14517.57 Da) but it needs to be further investigated as in the present work only male extracts were analysed at the LC-ESI FTMS.

### Comparison between sexes

**Figure 2:**
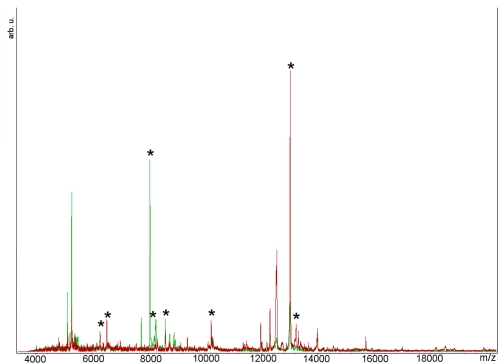
Average antennal mass spectra acquired through MALDI-TOF profiling on males (N = 16, green) and females (N = 9, red). Signals whose intensity resulted significantly different at the Kruskal Wallis test are marked with an asterisk.

A comparison between the antennal profiles of the two sexes was accomplished by calculating an average mass spectrum (using the program CPT) from the spectra obtained for each male (N = 9; 16 to 24 spectra for each antenna) and female antenna (N = 10; 12 to 27 spectra for each antenna) of sample A. The two protein profiles exhibited several differences, the most relevant of which are in the mass range between 8,000 and 9,000 Th, with the signal at 8,015 Th being very intense in males and almost missing in females, which exhibit instead an intense signal at 8,565 Th. Similar differences between sexes were also found for the profiling experiments performed on mosquitoes from sample B (one antenna from 16 males and one antenna from 9 females) (fig. 2). Male antenna spectra presented in fact a very intense ion at 8,015 which was absent in the females (P = 3.9×10^−4^, using the Kruskal–Wallis test), while these showed a signal more intense than in males as 8565 (P = 1.9×10^−2^). Other differences observed in both samples regarded an ion signal at 13006 Th being much more intense in female (P = 3.9×10^−4^ in sample B).

In both sexes, the spectra obtained for the mouthparts on sample A were quite similar to those obtained for the antennae and both differed considerably from those of the wings.

The comparison between the male average mouthparts and antennae protein patterns produced through CPT showed that most of the signals were in common, although especially in the 10,000–14,000 m/z range, most of the proteins produce more intense signals in the mouths. Mouthparts and antennae profiles differed considerably with respect to the wings (fig. S4), where the signal at 8,015 Th, the most intense in the mouths and in the antennae, was completely absent.

## Discussion

Good quality mass spectra were obtained both from profiling experiments performed directly on antennae of both males and females mosquitoes and from the MALDI MS analysis of antennal extracts. Although a consistent ion population was obtained by both approaches, MALDI MS direct profiling produced more satisfactory spectra, yielding more intense and numerous signals. These results show that MALDI MS profiling is suitable for the direct analysis of proteins of low and medium molecular weight in insect appendices, as small as mosquitoes antennae. Moreover, it is relevant to note that the straightforward preparation of the biological samples necessary for MALDI MS profiling allows to perform with a modest effort a high number of replicates necessary for statistical analyses. For insect species of small size, this constitutes an enormous advantage over traditional methods of protein expression analysis, where studies are commonly based on samples prepared from pools of hundreds to thousand specimens.

Previous gene expression studies have targeted OBPs genes expression in *An. gambiae* antennae [4], [6]. To verify whether the protein profiles obtained through the MALDI MS analysis include OBPs, we first performed tow-down experiments on antennal proteins and then a classic proteomic bottom-up experiment through MS and MS/MS analysis of male antennal proteins, separated on a 2D gel and digested *in-situ*. Although, a big effort was necessary to obtain enough tissue to carry out this latter approach (i.e. 400 male antennae were necessary for this preliminary experiment), this analysis allowed to confirm the presence of one OBP (i.e. OBP-9), previously identified on the basis of its accurate molecular weight and of a short sequence stretch obtained through the top-down experiment The identification of other proteins, however, was not possible due to low protein concentration in the 2D gel and would need large antennae samples to be completed. Hence, it is important to highlight that, despite the rapidity of acquisition of the spectra with direct profiling, significant effort is needed to identify the proteins present in the spectra. A second constrain in the profiling experiments is represented by the difficulty in matching the registered signals with the theoretical molecular weight of hypothetical proteins, predicted through genome annotations and/or EST libraries. Such discrepancy may have several causes, including polymorphism in the protein sequence between the populations/strains studied by each method, incorrect genome annotation, redundancy or errors in the aminoacid sequences, possible post-translational modifications and, in the case of secreted proteins, discrepancies between the predicted and the actual size of the clipped signal peptide. High resolution mass spectrometry could greatly help in finding the right match; nonetheless further structural characterization analyses, like partial *de-novo* ones may be required to confirm identification. In our work, the top-down approach utilised allowed the identification of two proteins (i.e. OBP9 and **gi|118781948**) present in the antennae. However, the high complexity of the antennal extract mixtures and the low concentration of single proteins in the extract limit the application of top-down experiments.

Despite the above technical constraints in the full identification of the antennal proteins (which was beyond the scope of this work), the results obtained show the suitability of the MALDI MS profiling for the detection of proteins directly from body parts of single insect specimens. The bottom-up approach, based on peptide fingerprinting and MS/MS experiments on peptides obtained from enzymatic digestions of proteins, is a classical strategy for protein identification and is currently in progress in our laboratory, to further extend the list of the proteins detected in the extracts and in the intact antennae. This procedure indeed proved successful in identifying OBPs in the silkmoth (*Bombyx mori*) and in the honeybee, (Dani et al., submitted).

The results obtained also highlight significant differences in the protein profiles between the two sexes of *An. gambiae*. The most relevant difference is found in the mass peak at 8,015 Th, which was attributed to a protein encoded by a gene reported as gi|118781948 (**XP_311966**). To our knowledge, this gene has never been reported as differentially expressed in the antenna of males and females *An. gambiae*. Moreover, none of the signals differentiating the two sexes by MALDI profiling corresponded to the theoretical molecular weight of proteins encoded by genes showing differential expression levels based on genomic analyses [4], [6]. This suggests that such proteomic analyses might complement to a significant extent, the results of genomic-based, differential gene expression analyses aimed at clarify molecular differences in odour perception between sexes.

The overall results show that MALDI MS profiling is a powerful technique for the analysis of small and medium MW proteins in small insect appendices, and allows to straightforwardly obtain data for several single specimens. Therefore, the proposed approach can represent a useful complement to the genomic approaches commonly used for this kind of studies, by allowing to directly analyse the phenotypic expression of OBPs instead of their transcription patterns.

## Supporting Information

Figure S1 MALDI imaging of an Anopheles gambiae male antenna. MALDI-TOF imaging MS experiment on male antennae from An. gambiae. Protein images were obtained by setting the intensity scale between 5% (minimum intensity) and 100% (full intensity threshold). Images have also been normalized using Flex Imaging 2.0 and setting the Ymean/Ymax threshold at 0.02. Ion images of four proteins are reported: 8015 Th, 8565 Th, 11941 Th and 13936 Th respectively in panel A, B, C, and D, showing a different distribution across the antenna.
(0.23 MB TIF)

Figure S2 Identification of OBP-9 through analysis performed on an LTQ Orbitrap mass spectrometer. For the ion signal corresponding to OBP-9, the figure reports the multicharged ions (A), a zoom for the z = 9 ions (B), the deconvoluted spectrum (C), the monoisotopic mass (calculated by using the Extract Tool integrated in the Excalibur 2.0 Software by considering the multicharged ion at z = 9) (D) and the MS/MS spectrum (E) on the ion at 1,394.59 Th (z = 10) which produced three internal ion fragments (z = 9) corresponding to the successive loss of two valine residues.
(0.42 MB TIF)

Figure S3 MS/MS spectrum resulting from the top-down experiment on the precursor ion at 1,604.12 Th (z = 5) and its interpretation. The search for the reported stretch in the An. gambiae genome found a hit for a hypothetical protein (gi|118781948) having a theoretical monoisotopic mass of 8,010.58 Da, which was in agreement with the experimental one (8,010.53 Da).
(0.26 MB TIF)

Figure S4 Comparison between male antenna (green) and wing (red) mass spectra. Average mass spectra resulting from the analysis through ClinProt Tool on the spectra obtained through MALDI-TOF profiling.
(0.15 MB TIF)

Text S1 Protein homogenate characterization by MALDI-TOF MS and LC-ESI FTMS
(0.03 MB DOC)
