# Supplementary figures and images for: Exploring Proteins in Anopheles gambiae Male and Female Antennae through MALDI Mass Spectrometry Profiling
Source: PLoS One. 2008 Jul 30;3(7):e2822. doi: 10.1371/journal.pone.0002822 (PMC2474704; doi:10.1371/journal.pone.0002822)

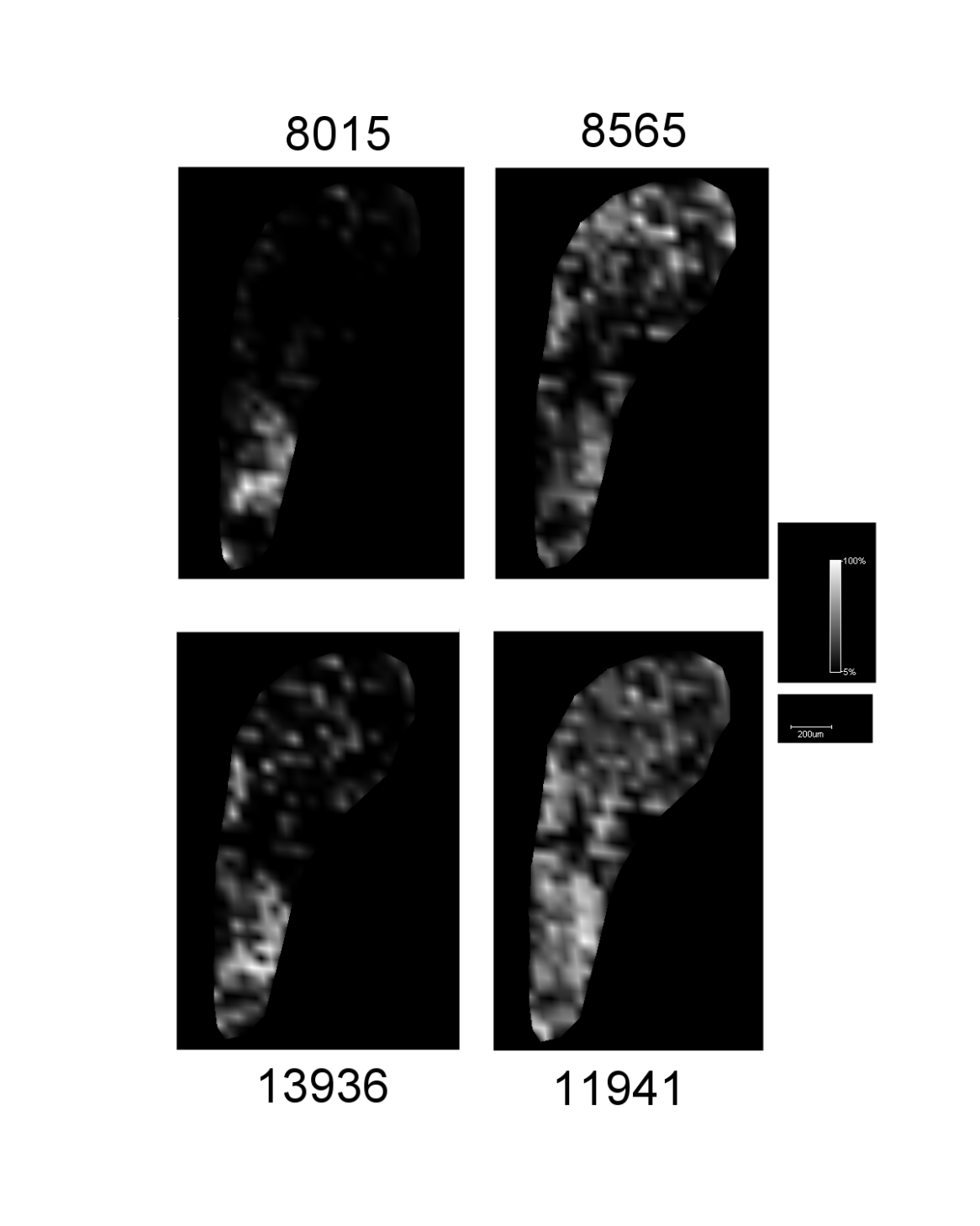

Supplement: Figure S1 — MALDI imaging of an Anopheles gambiae male antenna. MALDI-TOF imaging MS experiment on male antennae from An. gambiae. Protein images were obtained by setting the intensity scale between 5% (minimum intensity) and 100% (full intensity threshold). Images have also been normalized using Flex Imaging 2.0 and setting the Ymean/Ymax threshold at 0.02. Ion images of four proteins are reported: 8015 Th, 8565 Th, 11941 Th and 13936 Th respectively in panel A, B, C, and D, showing a different distribution across the antenna. (0.23 MB TIF) [file pone.0002822.s001.tif]

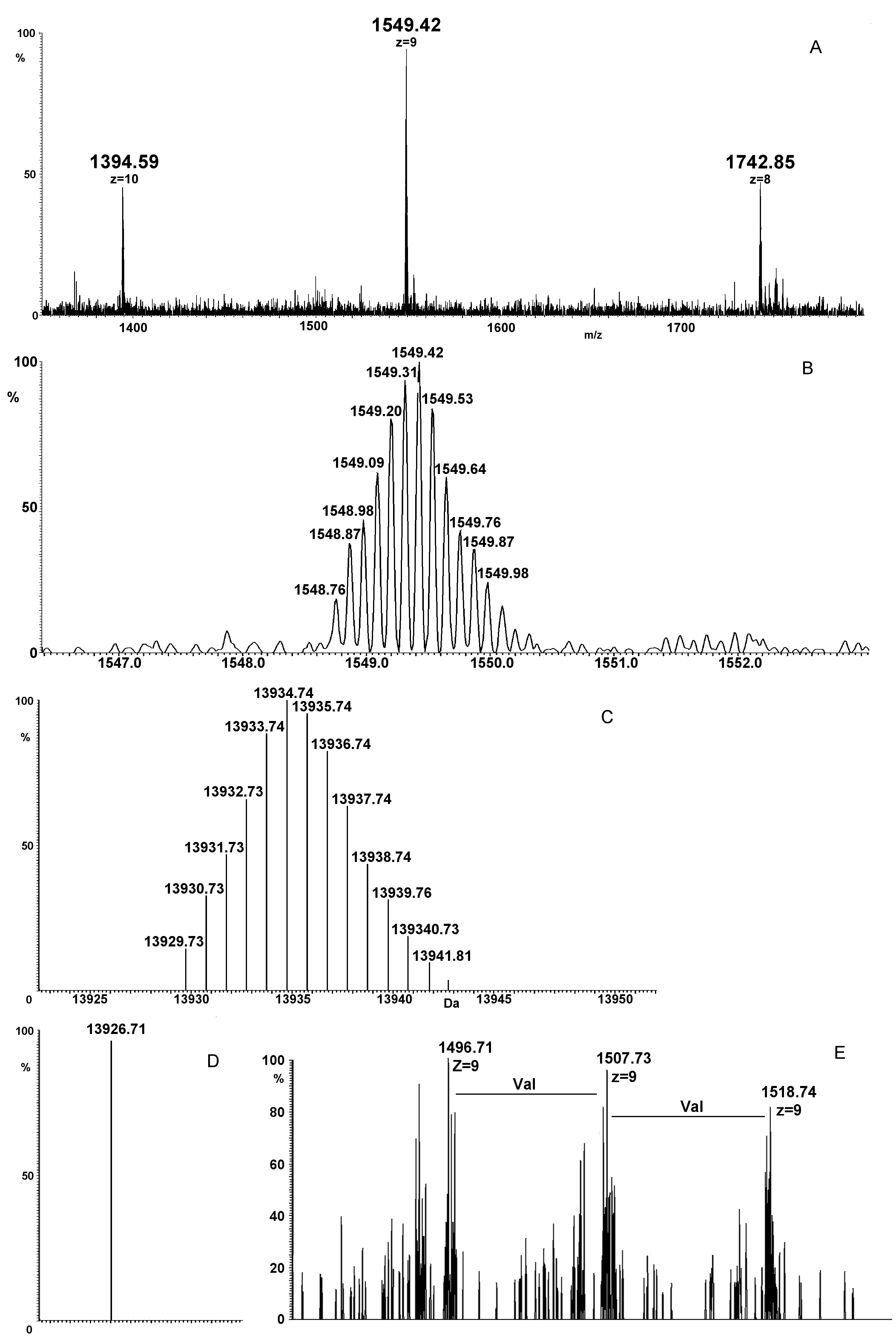

Supplement: Figure S2 — Identification of OBP-9 through analysis performed on an LTQ Orbitrap mass spectrometer. For the ion signal corresponding to OBP-9, the figure reports the multicharged ions (A), a zoom for the z = 9 ions (B), the deconvoluted spectrum (C), the monoisotopic mass (calculated by using the Extract Tool integrated in the Excalibur 2.0 Software by considering the multicharged ion at z = 9) (D) and the MS/MS spectrum (E) on the ion at 1,394.59 Th (z = 10) which produced three internal ion fragments (z = 9) corresponding to the successive loss of two valine residues. (0.42 MB TIF) [file pone.0002822.s002.tif]

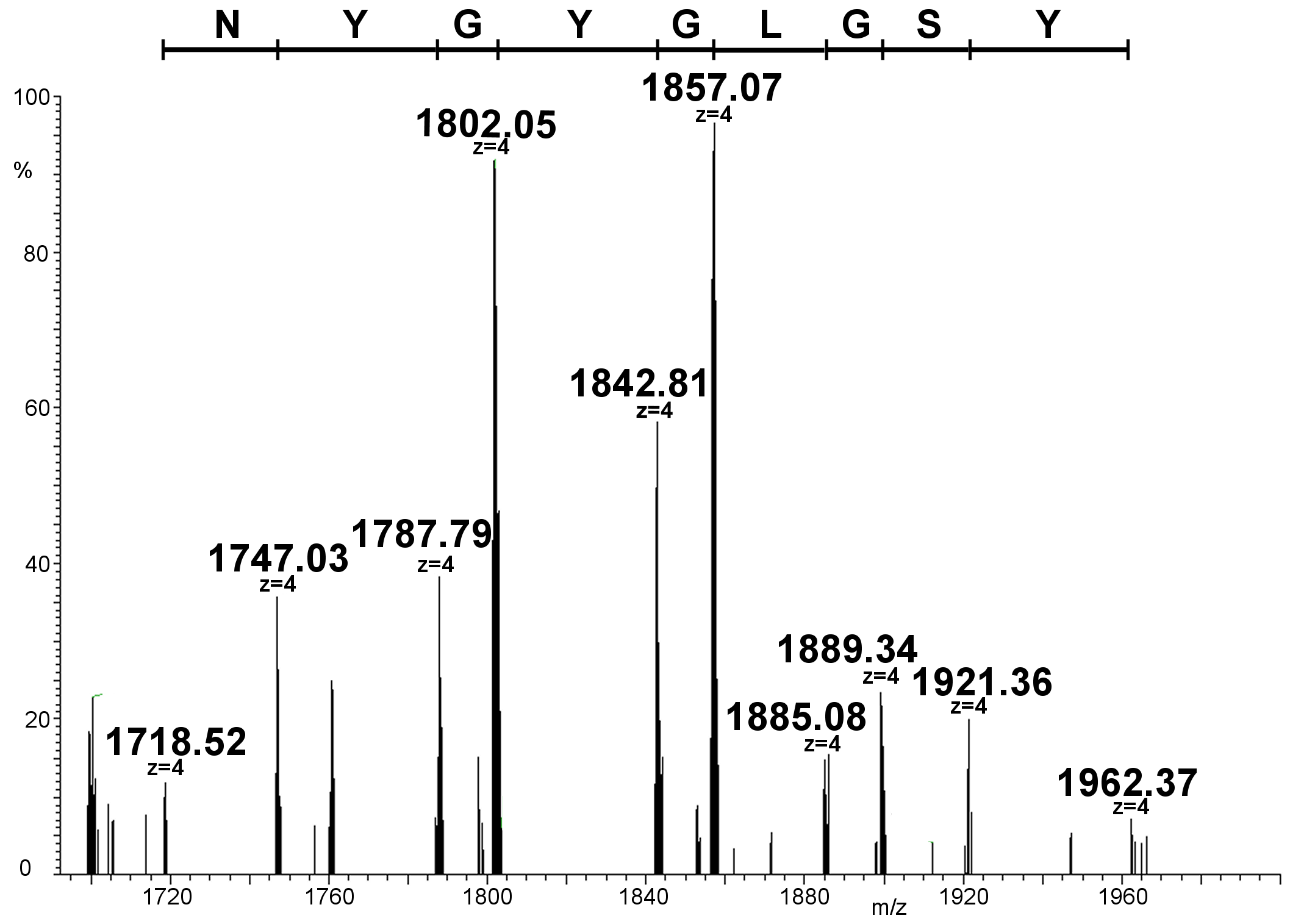

Supplement: Figure S3 — MS/MS spectrum resulting from the top-down experiment on the precursor ion at 1,604.12 Th (z = 5) and its interpretation. The search for the reported stretch in the An. gambiae genome found a hit for a hypothetical protein (gi|118781948) having a theoretical monoisotopic mass of 8,010.58 Da, which was in agreement with the experimental one (8,010.53 Da). (0.26 MB TIF) [file pone.0002822.s003.tif]

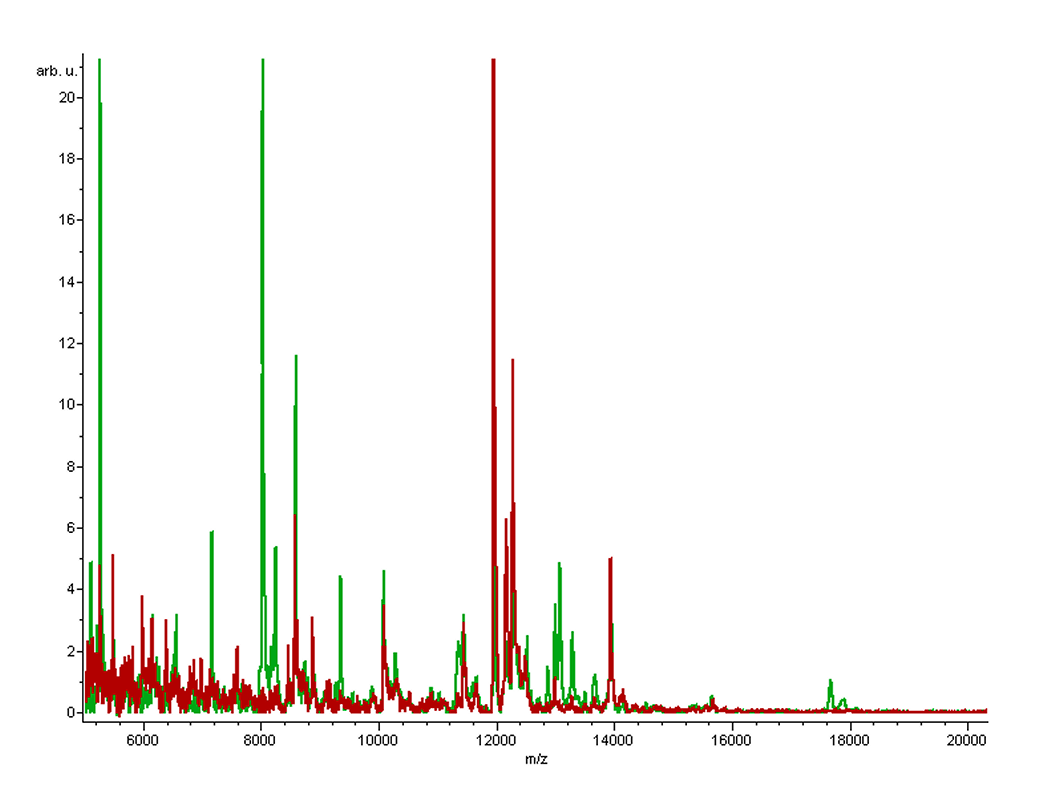

Supplement: Figure S4 — Comparison between male antenna (green) and wing (red) mass spectra. Average mass spectra resulting from the analysis through ClinProt Tool on the spectra obtained through MALDI-TOF profiling. (0.15 MB TIF) [file pone.0002822.s004.tif]
