# Supplementary material for: Exploring Proteins in Anopheles gambiae Male and Female Antennae through MALDI Mass Spectrometry Profiling
Source: PLoS One. 2008 Jul 30;3(7):e2822. doi: 10.1371/journal.pone.0002822 (PMC2474704; doi:10.1371/journal.pone.0002822)
Supplement: Text S1 — Protein homogenate characterization by MALDI-TOF MS and LC-ESI FTMS (0.03 MB DOC) [file pone.0002822.s005.doc]

**Supplemental text**

# Protein homogenate characterization by MALDI-TOF MS and LC-ESI FTMS

For the protein registered at the LC-ESI FT MS as having an average mass of 13,935.74 Da and a monoisotopic mass of 13,926.71 Da, the top-down experiment was performed on the multicharged ion at m/z=1394.59 (fig. S2A) and produced three fragments at z=9 (fig. S2E). The nine charge fragment at 1,518.74 Th corresponds to a deconvoluted mass of 13,659.66 Da, matching with the molecular weight expected for the sequence 3-139 of the processed OBP-9 protein (20-139 of the unprocessed precursor). Moreover, the difference between the masses of the multicharged ion at z=10 (1,394.59 Th) and the first produced ion at z=9 (1,518.74 Th), may correspond to the loss of glutamic acid and phenyl alanine residues (EF, 276.21 Da) which indeed are the first two amino acids in the mature OBP-9. Finally, the m/z values of the fragment ions at 1,518.74 , 1,507.73 and 1,496.71 Th (z=9), could be explained as sequential loss of two valine (V) residues, which are the following amino acids after EF.

For the protein having a monoisotopic mass of 8,010.53 Da, a top-down experiment (fig. S3) was performed on the precursor ion at 1,604.12 Th (z=5). Then ion fragments (z=4) were detected from which we could derive the sequence YSG(I/L)GYGYN. Blasting both YSGIGYGYN and YSGLGYGYN as well as their reverse sequence (<http://130.14.29.110/BLAST/>), using “[Search for short, nearly exact matches](http://130.14.29.110/BLAST/Blast.cgi?CMD=Web&LAYOUT=TwoWindows&AUTO_FORMAT=Semiauto&ALIGNMENTS=50&ALIGNMENT_VIEW=Pairwise&CLIENT=web&DATABASE=nr&DESCRIPTIONS=100&ENTREZ_QUERY=(none)&EXPECT=20000&FORMAT_OBJECT=Alignment&FORMAT_TYPE=HTML&GAPCOSTS=9+1&I_THRESH=0.005&MATRIX_NAME=PAM30&NCBI_GI=on&PAGE=Proteins&PROGRAM=blastp&SERVICE=plain&SET_DEFAULTS.x=24&SET_DEFAULTS.y=10&SHOW_OVERVIEW=on&WORD_SIZE=2&END_OF_HTTPGET=Yes&SHOW_LINKOUT=yes&GET_SEQUENCE=yes&SEARCH_NAME=short_bp)” against *Anopheles* genome, we could find a hit (gi|118781948) for YSGLGYGYN sequence (Y32-N40), having a molecular weight of 11,156.30 Da. Assuming a cleavage of the corresponding polypeptide between aa 29 and 30, the mature protein including aa 30-108 would weight 8,015.57 Da. A perfect agreement was found between the monoisotopic experimental and calculated MW (8,010.53 Da and 8,010.57 Da respectively). Finally the MWs resulting from the deconvolution of the multicharged ion fragments matched those expected for the amino acidic sequences 32-108, 33-108, 34-108, 35-108, 36-108, 37-108, 38-108, 39-108, 40-108 of the protein.

**Supplemental figure 1**

**Fig. S1. MALDI imaging of an *Anopheles gambiae* male antenna.** MALDI-TOF imaging MS experiment on male antennae from *An. gambiae*. Protein images were obtained by setting the intensity scale between 5% (minimum intensity) and 100% (full intensity threshold). Images have also been normalized using Flex Imaging 2.0 and setting the Ymean/Ymax threshold at 0.02. Ion images of four proteins are reported: 8015 Th, 8565 Th, 11941 Th and 13936 Th respectively in panel A, B, C, and D, showing a different distribution across the antenna.

**Supplemental figure 2**

**Fig. S2. Identification of OBP-9 through analysis performed on an LTQ Orbitrap mass spectrometer.** For the ion signal corresponding to OBP-9, the figure reports the multicharged ions (A), a zoom for the z=9 ions (B), the deconvoluted spectrum (C), the monoisotopic mass (calculated by using the Extract Tool integrated in the Excalibur 2.0 Software by considering the multicharged ion at z=9) (D) and the MS/MS spectrum (E) on the ion at 1,394.59 Th (z=10) which produced three internal ion fragments (z=9) corresponding to the successive loss of two valine residues.

**Supplemental figure 3**

**Fig. S3. MS/MS spectrum resulting from the top-down experiment on the precursor ion at 1,604.12 Th (z=5) and its interpretation.** The search for the reported stretch in the *An. gambiae* genome found a hit for a hypothetical protein (gi|118781948) having a theoretical monoisotopic mass of 8,010.58 Da, which was in agreement with the experimental one (8,010.53 Da).

**Supplemental figure 4**

**Fig. S4. Comparison between male antenna (green) and wing (red) mass spectra.** Average mass spectra resulting from the analysis through ClinProt Tool on the spectra obtained through MALDI-TOF profiling.
